# Sex disparity in the management and outcomes of dyslipidemia of diabetic patients in the Arabian Gulf: findings from the CEPHEUS study

**DOI:** 10.1186/s12944-018-0667-y

**Published:** 2018-02-05

**Authors:** Ibrahim Al-Zakwani, Fatma Al-Mahruqi, Khalid Al-Rasadi, Abdullah Shehab, Wael Al Mahmeed, Mohammed Arafah, Ali T. Al-Hinai, Omer Al Tamimi, Mahmoud Al Awadhi, Raul D. Santos

**Affiliations:** 1 Department of Pharmacology & Clinical Pharmacy, College of Medicine & Health Sciences, Sultan Qaboos University, PO Box 38, PC-123, Al-Khodh, Sultanate of Oman; 2 Gulf Health Research, Muscat, Oman; 3 College of Medicine & Health Sciences, Sultan Qaboos University, Muscat, Oman; 4 Department of Biochemistry, Sultan Qaboos University Hospital, Muscat, Oman; 5 UAE University, Al-Ain, UAE; 6 Heart and Vascular Institute, Cleveland Clinic, Abu Dhabi, UAE; 7 King Saud University Hospital, Riyadh, Saudi Arabia; 8 Ministry of Health, Muscat, Oman; 9 Hamad Medical Corporation, Doha, Qatar; 10 Al-Amiri Hospital, Kuwait City, Kuwait; 11 Lipid Clinic Heart Institute (InCor), University of Sao Paulo Medical School Hospital, Sao, Paulo, Brazil

**Keywords:** Cardiovascular disease, Low density lipoprotein cholesterol, Apolipoprotein B, Sex, Arabs, Middle East

## Abstract

**Background:**

Little is known about sex gap in the management and outcomes of dyslipidemia among diabetics in the Arabian Gulf. The aim if this study was to determine sex differences in the management and outcomes of dyslipidemia in diabetic patients in the Arabian Gulf.

**Methods:**

This study was derived from the Centralized Pan-Middle-East Survey on the management of hypercholesterolemia. Patients recruited were aged ≥18 years on lipid lowering drugs for ≥3 months (stable medication for ≥6 weeks). Outcomes were based on the joint Consensus Statement of the American Diabetes Association and American College of Cardiology Foundation. Analyses were performed using univariate and multivariate logistic regression techniques.

**Results:**

The mean age of the cohort (*n* = 3336) was 57 ± 11 years and 45% (*n* = 1486) were females. Females were less likely to be on rosuvastatin (7.6% vs 12%; *P* < 0.001), atorvastatin (41% vs 46%; *P* = 0.005) and combination hypolipidemic therapy (5.6% vs 2.8%; *P* < 0.001) but more likely to be on simvastatin (51% vs 39%; *P* < 0.001) than males. Females, especially those with very high atherosclerotic cardiovascular disease (ASCVD) risk status, were also less likely to achieve LDL-cholesterol [adjusted odds ratio (aOR), 0.58; 95% confidence interval (CI): 0.40–0.86; *P* = 0.006], non-HDL-cholesterol [aOR, 0.68; 95% CI: 0.46–0.99; *P* = 0.048] and apolipoprotein B [aOR, 0.64; 95% CI: 0.44–0.92; *P* = 0.016] lipid targets.

**Conclusions:**

Diabetic women were less likely to be on optimal hypolipemic therapy and consequently less likely to attain lipid goals compared to men. This shows a sex gap on dyslipidemia treatment in the region. Diabetic women with very high ASCVD risk status need to be aggressively treated to lower their risk of cardiovascular events.

## Background

Type 2 diabetes mellitus is a global public health issue that affects around 8.5% of adult population according to the World Health Organization [1]. This problem is particularly alarming in the Middle East region where the average prevalence is more than twice (14.8–20%) of Western countries [2]. The prevalence of atherosclerotic cardiovascular disease (ASCVD) and its associated morbidity and mortality are substantially higher among diabetics than non-diabetics [3]. Furthermore, women with diabetes have considerably higher risk of coronary heart disease (CHD) mortality compared to men, even after adjusting for confounding factors [4, 5].

Dyslipidemia management is an important prevention strategy to reduce cardiovascular risk in both sexes [6–9]. However, despite equitable access to lipid lowering therapy, cardiovascular disease studies have shown that women are less likely to attain their optimal lipid goals as compared to men [10–17]. Despite the high prevalence of dyslipidemia [18] and diabetes [2] in the Arabian Gulf region, there are currently limited data assessing sex disparity gaps in management and outcomes of dyslipidemia among diabetics in this region. Hence, the objective of this study was to determine sex differences in management and outcomes among diabetic patients in the Arabian Gulf.

## Methods

The details of this study have been previously described [19]. Briefly, the CEPHEUS study was a multi-center non-interventional survey of patients on lipid lowering drugs (LLDs) in six Arabian Gulf countries (Saudi Arabia, United Arab Emirates, Oman, Qatar, Bahrain and Kuwait). A total of 5457 patients were enrolled in this survey from outpatient clinics by 177 specialists and primary care physicians. However, this sub study included only those that were diabetic (*n* = 3336) and had non-missing information on sex. The study was conducted between November 2009 and July 2010. The inclusion criteria were: patients ≥18 years of age taking LLDs for ≥3 months and with no dose change for a minimum of 6 weeks.

A fasting blood sample was taken from each subject for measurement of total cholesterol (TC), high-density lipoprotein cholesterol (HDL-C), low-density lipoprotein cholesterol (LDL-C), triglycerides (TG), apolipoprotein B (ApoB), glucose and glycated hemoglobin A1c (Hba1c). All blood samples were tested at the King Faisal specialist Hospital and Research Centre (Riyadh, Saudi Arabia). All laboratory tests underwent internal and external quality control checks.

Study subjects were checked for statin use, type, and specifically the use of high doses of atorvastatin (40–80 mg) and rosuvastatin (20-40 mg). Statin combination was defined as statin prescription along with the addition of other LLDs. Other LLDs included fibrates (benzafibrate, fenofibrate, gemfibrozil), bile acid sequestrant (colestipol) and ezetimibe.

Criteria for ASCVD risk status was derived from the National Lipid Association (NLA) recommendations for patient-centered management of dyslipidemia Part 1–Executive Summary [20]. High-risk group included diabetic patients (type 1 or 2) with 0/1 other major ASCVD risk factor or LDL-C ≥ 5.02 mmol/L (190 mg/dL; severe hypercholesterolemia). The very high-risk group included ASCVD or diabetes mellitus with ≥2 other major ASCVD risk factors. Low HDL-C was defined as levels of 1.0 mmol/L (< 40 mg/dL) for men and 1.3 mmol/L (< 50 mg/dL) for women. Therapeutic lipoprotein targets for the high-risk patients were LDL-C < 2.6 mmol/L (100 mg/dL), ApoB < 0.90 g/L and non-HDL-C < 3.3 mmol/L (130 mg/dL). For the highest risk group, therapeutic lipoprotein targets were LDL-C < 1.8 mmol/L (70 mg/dL), ApoB < 0.80 g/L and non-HDL-C < 2.6 mmol/L (100 mg/dL) [20].

### Statistical analysis

For categorical variables, frequencies and percentages were reported and differences between groups were analyzed using Pearson’s χ2 tests (or Fisher’s exact tests for cells < 5). For continuous variables, mean and standard deviation were used to summarize the data. Analyses were performed using Student’s t-tests. The association between LDL-C, non-HDL-C and ApoB goal attainment and sex was evaluated using multivariate logistic regression models adjusted for age, body mass index (BMI), smoking status, metabolic syndrome, baseline TG and LDL-C as well as statins prescribed (simvastatin or atorvastatin and rosuvastatin) and the associated dose strengths of the latter. The goodness-of-fit of the logistic model was examined using the Hosmer and Lemeshow goodness-of-fit statistic [21]. The Hosmer and Lemeshow test analyses the actual versus the predicted responses; theoretically, the observed and expected counts should be close. Based on the χ [2] distribution, a Hosmer and Lemeshow statistic with a *P* > 0.05 is considered a good fit. An a priori two-tailed level of significance was set at 0.05. Statistical analysis was carried out using STATA version 13.1 (STATA Corporation, College Station, TX, USA).

## Results

Table 1 shows demographic and clinical characteristics of the whole cohort and according to sex. The overall mean age was 57 ± 11 years and 45% (*n* = 1486) were females. The average BMI was 32 kg/m^2^ and 55% (*n* = 1843) were obese. The proportion of patients with coronary heart disease, metabolic syndrome and hypertension were 30% (*n* = 999), 76% (*n* = 2437) and 70% (*n* = 2330), respectively. Most patients (83%; *n* = 2755) had very high ASCVD risk status. The majority (95%; *n* = 3160) was on statin monotherapy. Patients on statin combination and other LLDs constituted only 4.4% (*n* = 145) and 0.9% (*n* = 31), respectively.

**Table 1:** Demographic and clinical characteristics of the CEPHEUS diabetic cohort stratified by sex (*N* = 3336)

Characteristic, mean ± SD unless specified otherwise	All (*N* = 3336)	Female (*n* = 1486) 45%	Male Female (*n* = 1850) 55%	*P-value*
Demographic				
Gulf citizen, *n* (%)	2667 (80%)	1337 (90%)	1330 (72%)	< 0.001
Age, years	57 ± 11	57 ± 10	57 ± 11	0.883
Weight, kg	83 ± 18	80 ± 17	85 ± 18	< 0.001
Waist circumference, cm	105 ± 14	104 ± 14	105 ± 14	0.097
BMI, kg/m^2^	32 ± 7	34 ± 7	30 ± 6	< 0.001
BMI > 30 kg/m^2^, *n* (%)	1843 (55%)	1018 (69%)	825 (45%)	< 0.001
Clinical, n (%)				
Current smoker	381 (11%)	21 (1.4%)	360 (19%)	< 0.001
Hypertension	2330 (70%)	1031 (69%)	1299 (70%)	0.601
Coronary heart disease	999 (30%)	251 (17%)	748 (40%)	< 0.001
PVD	101 (3.0%)	35 (2.4%)	66 (3.6%)	0.042
Cerebrovascular disease	135 (4.1%)	44 (3.0%)	91 (4.9%)	0.004
Metabolic syndrome	2437 (76%)	1172 (81%)	1265 (72%)	< 0.001
Very high ACSVD risk	2755 (83%)	1110 (75%)	1645 (89%)	< 0.001
HbA1c, %	8.6% ± 3.7%	8.7% ± 3.5%	8.5% ± 3.9%	0.001
HbA1c < 7%	863 (26%)	382 (26%)	481 (26%)	0.851
Dyslipidemia therapy, *n* (%)				
Statin monotherapy	3160 (95%)	1439 (97%)	1721 (93%)	< 0.001
Statin combination	145 (4.4%)	41 (2.8%)	104 (5.6%)	< 0.001
Others^a^	31 (0.9%)	6 (0.4%)	25 (1.4%)	0.005
Lipid levels on treatment				
TC	4.28 ± 1.09	4.47 ± 1.08	4.13 ± 1.08	< 0.001
LDL-C	2.50 ± 0.90	2.59 ± 0.89	2.42 ± 0.90	< 0.001
HDL-C	1.16 ± 0.31	1.28 ± 0.31	1.06 ± 0.27	< 0.001
ApoB, g/L	0.91 ± 0.27	0.93 ± 0.27	0.89 ± 0.27	< 0.001
Non-HDL-C	3.13 ± 1.06	3.19 ± 1.08	3.07 ± 1.05	0.001
TG	1.79 ± 1.37	1.73 ± 1.30	1.84 ± 1.42	0.021

*SD*, standard deviation; *BMI*, body mass index; *PVD*, peripheral vascular disease; *TC*, total cholesterol; *LDL-C*, low-density lipoprotein cholesterol; *HDL-C*, high-density lipoprotein cholesterol; *ApoB*, apolipoprotein B; *TG*, triglyceride

^a^Others included fibrates (benzafibrate, fenofibrate, gemfibrozil), bile acid sequestrant (colestipol) and ezetimibe

Percentages might not add up to 100% due to missing information as shown below

Age (*n* = 8), weight (*n* = 2), waist (*n* = 69), BMI (*n* = 10), HbA1c (*n* = 13), metabolic syndrome (*n* = 131), systolic BP (*n* = 6), diastolic BP (*n* = 6) and BP (*n* = 6) were missing in some subjects

Table 1 also shows that females had a greater prevalence of obesity (*P* < 0.001) and the metabolic syndrome (*P* < 0.001). They also had higher TC (*P* < 0.001), LDL-C (*P* < 0.001), non-HDL-C (*P* = 0.001) and ApoB (*P* < 0.001) concentrations. On the other hand, males had a greater prevalence of smoking (*P* < 0.001), coronary heart (*P* < 0.001), peripheral (*P* = 0.042) and cerebrovascular (*P* = 0.004) diseases. Of importance, every 3 in 4 women were considered as being at very high ASCVD risk, however this prevalence was higher in men (roughly 9 in 10 individuals, *P* < 0.001).

**Fig. 1:**
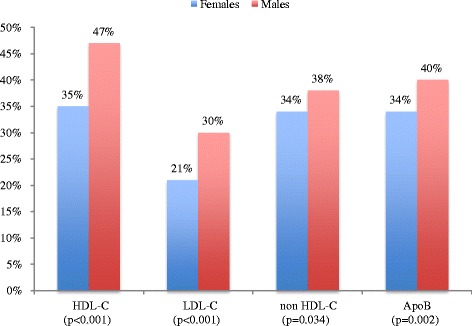
Lipid target achievements (LDL-C, non HDL-C and Apo B) of very high risk atherosclerotic vascular (ASCVD) diabetic patients stratified by sex (females *n* = 1110), males *n* = 1645). HDL-C, high-density lipoprotein cholesterol; LDL-C, low-density lipoprotein cholesterol; Apo B, apolipoprotein B

**Fig. 2:**
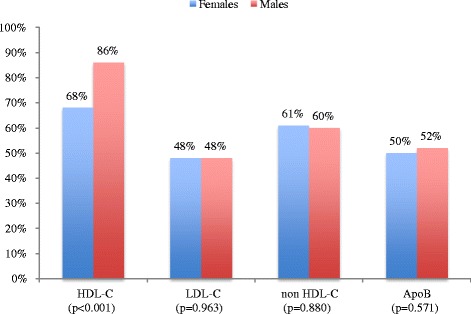
Lipid target achievements (HDL-C, LDL-C, non HDL-C and Apo B) in high risk atherosclerotic vascular (ASCVD) diabetic patients stratified by sex (females *n* = 376, males *n* = 205). HDL-C, high-density lipoprotein cholesterol; LDL-C, low-density lipoprotein cholesterol; Apo B, apolipoprotein B

As shown in Fig. 1, when compared to males, females at highest ASCVD risk category had a greater proportion of low HDL-C levels (35% vs 47%; *P* < 0.001) and achieved less LDL-C (21% vs 30%; *P* < 0.001), non-HDL-C (34% vs 38%; *P* = 0.034) and ApoB (34% vs 40%; *P* = 0.002) goals. Figure 2 shows that females at the high ASCVD risk category, apart from lower HDL-C (68% vs 86%; *P* < 0.001) levels, there were no significant differences in lipid target achievements.

Table 1 shows that women were more likely to be on statin monotherapy compared to men (*P* < 0.001). However, they were less likely to be on more efficacious lipid lowering therapies like atorvastatin (41% vs 46%; *P* = 0.003) and rosuvastatin (7.2% vs 11%; *P* < 0.001) than males. On the same token, females with *very high* ASCVD risk status were less likely to be associated with use of high doses of atorvastatin (40 and 80 mg) compared to males (23% vs 29%; *P* = 0.035); however, no significant differences were noted regarding the use of 20 and 40 mg doses of rosuvastatin (combined) (39% vs 31%; *P* = 0.211).

In those with *high* ASCVD risk status, no differences were noted between the sexes regarding the use of high doses of both atorvastatin (10% vs 11%; *P* = 0.968) and rosuvastatin (38% vs 13%; *P* = 0.154; *power* = 24%). On the contrary, female sex was associated with a greater use of simvastatin (51% vs 41%; *P* < 0.001). Furthermore, females were also less likely to be prescribed statin drug combination (2.8% vs 5.6%; *P* < 0.001) and other LLDs (0.4% vs 1.4%; *P* = 0.005).

Table 2 shows the association between LDL-C, non-HDL-C and ApoB goal attainment and sex using multivariate logistic regression models adjusting for confounders. The results indicated that females with very high ASCVD risk were less likely to achieve LDL-C [adjusted odds ratio (aOR), 0.58; 95% confidence interval (CI): 0.40–0.86; *P* = 0.006], non-HDL-C [aOR, 0.68; 95% CI: 0.46–0.99; *P* = 0.048] and ApoB [aOR, 0.64; 95% CI: 0.44–0.92; *P* = 0.016] goal attainments. Of note, high TGs were also inversely associated with non-HDL-C [aOR, 0.16; 95% CI: 0.11–0.24; *P* < 0.001] and ApoB [aOR, 0.30; 95% CI: 0.22–0.41; *P* < 0.001] goal attainments. Of interest, simvastatin use in diabetics with very high ASCVD was also inversely associated with LDL-C [aOR, 0.49; 95% CI: 0.32–0.75; *P* = 0.001], non-HDL-C [aOR, 0.55; 95% CI: 0.37–0.83; *P* = 0.004] and ApoB [aOR, 0.53; 95% CI: 0.37–0.78; *P* = 0.001] goal attainments.

**Table 2:** The associations between LDL-C, non-HDL-C and Apo B goal attainment and sex, in diabetics with very high ASCVD risk status, adjusting for various other factors in the models, were performed using multivariate logistic regressions

Characteristic	LDL-C goal		Non-HDL-C goal		Apo B goal	
	aOR[95% CI]	*P-value*	aOR[95% CI]	*P-value*	aOR[95% CI]	*P-value*
Female	0.58 [0.40–0.86]	0.006	0.68 [0.46–0.99]	0.048	0.64 [0.44–0.92]	0.016
Age	1.01 [0.98–1.03]	0.217	1.01 [0.99–1.02]	0.558	1.00 [0.99–1.02]	0.587
BMI	1.00 [0.97–1.03]	0.734	1.00 [0.97–1.03]	0.984	1.00 [0.97–1.02]	0.860
Smoker	0.68 [0.41–1.12]	0.127	0.78 [0.47–1.30]	0.342	0.70 [0.43–1.13]	0.142
MetS	0.84 [0.57–1.23]	0.371	1.59 [1.06–2.39]	0.026	1.15 [0.78–1.68]	0.474
TG	0.86 [0.71–1.04]	0.127	0.16 [0.11–0.24]	< 0.001	0.30 [0.22–0.41]	< 0.001
LDL-C	1.00 [1.00–1.00]	0.857	1.00 [1.00–1.00]	0.420	1.00 [1.00–1.00]	0.127
Simvastatin	0.49 [0.32–0.75]	0.001	0.55 [0.37–0.83]	0.004	0.53 [0.37–0.78]	0.001
Statin dose	1.00 [1.00–1.00]	0.251	1.00 [1.00–1.00]	0.548	1.00 [1.00–1.01]	0.204

*LDL-C*, low-density lipoprotein cholesterol; non *HDL-C*, non high-density lipoprotein cholesterol; *Apo B*, apolipoprotein B; *ASCVD*, atherosclerotic vascular disease; *aOR*, adjusted odds ratio; *CI*, confidence interval; *BMI*, body mass index; *MetS*, metabolic syndrome; *TG*, triglycerides

The associations between LDL-C, non-HDL-C and ApoB goal attainment and sex were evaluated using multivariate logistic regression models adjusted for age, BMI, smoking status, MetS, baseline TG and LDL-C as well as statins prescribed (1 = simvastatin, 0 = atorvastatin and rosuvastatin) and the associated dose strengths of statins

In a secondary analysis (hypothesis generating), when we analyzed only those diabetics with very high ASCVD risk status and on atorvastatin (*N* = 1210), females were still less likely to attain HDL-C (33% vs 45%; *P* < 0.001) and LDL-C (24% vs 36%; *P* < 0.001) lipid goals. They were, however, no significant difference in lipid goal attainments for non-HDL-C (41% vs 45%; *P* = 0.192) and Apo B (43% vs 47%; *P* = 0.254). When the analysis was repeated for those diabetics with very high ASCVD risk status and on rosuvastatin (*N* = 284), there were no significant differences in goal attainment in all the lipid fractions HDL-C (38% vs 46%; *P* = 0.224), LDL-C (33% vs 44%; *P* = 0.102), non-HDL-C (45% vs 49%; *P* = 0.544) and Apo B (38% vs 48%; *P* = 0.126). However, these results should be interpreted with caution due low study power (low sample size). Furthermore, since the CEPHEUS study did not capture doses of the statins used, a finding of lower effectiveness in statins could not be made conclusively.

## Discussion

This study, the first performed in the Arabian Gulf, showed that very high ASCVD risk diabetic men and women present with low rates of attaining proposed lipid and ApoB goals to prevent cardiovascular events. This finding however, was significantly greater in women. In addition, despite equitable access to medications, women were less likely to be prescribed more intensive lipid lowering therapy compared to men. Therefore, a clear sex gap in dyslipidemia management was demonstrated in the region.

Although women with diabetes mellitus have an increased risk of cardiovascular morbidity and mortality compared with diabetic men [4, 5], the present study revealed that women were undertreated and less likely to attain the recommended lipid and ApoB goals than their male counterparts. Similar findings have been reported elsewhere [12, 22, 23].

The reasons for the gender disparity in the management of dyslipidemia are not totally clear. However, several possible explanations have been put forward. It’s reported that, in general, women are less concerned about their health and may not raise their symptoms with their physicians [24]. Goldberg et al. also reported that women may experience CVD symptoms that are atypical and different from those of men and hence may not discuss their encounters with their physicians and consequently their CVD symptoms may not be further evaluated [25]. Physicians have also been reported to perceive women at lower risk than men despite having similar CHD risk equivalents [26].

This study has also demonstrated that lipid treatment goals, especially for those with diabetes and very high ASCVD risk status, are significantly lower in females compared to males. These findings are consistent with previous studies [10–17]. In an earlier published study from the CEPHEUS project, it also documented that women were less likely to attain their lipid target achievements in high and very high ASCVD risk patients in the Arabian Gulf [10]. However, the current study aimed to look at gender disparity only in dyslipidemia management and lipid outcomes in the diabetic population with CHD. Gender differences in lipid goal attainment rates between men and women have been explained by differences in socioeconomic status, cardiovascular co-morbidities and associated risk factors, baseline lipid level, and the dosage of statin treatment [15]. In the Lipid Treatment Assessment Panel-2 study (LTAP-2), diabetes, hypertension and the presence of the metabolic syndrome were associated with a greater chance of failing LDL-C goal success in women, while in men it was only diabetes [12]. Gene-gender interactions may also contributed to gender disparities in lipid goal attainment [27]. For example, an apolipoprotein E (ApoE) polymorphism, in respect to statin therapy, has been found to be different between men and women [28].

In a review paper by Banach and colleagues [29], they concluded that statin non-adherence may be the main cause of inadequate LDL-C reduction. Statin associated muscle symptoms have been reported to be the most common cause of statin discontinuation or dose reduction [30]. In a metanalysis on gender and racial disparities to statin therapy, Lewey et al. [31] reported that women and non-white patients were at an increased risk for non-adherence to statin medication. However, in this diabetic cohort based on survey questions (*‘I always take my medication to lower cholesterol every day’*), there were no significant differences in adherence to statin medication between males and females (87% vs 89%; *P* = 0.221).

In this study, in the very high ASCVD category, most men and women were not in use of elevated doses of potent statins and lipid lowering drug associations however; this finding was more frequent in the latter. On the same token, women were more frequently receiving simvastatin, a less efficacious statin, compared to men who were receiving more potent atorvastatin and rosuvastatin. Indeed, the use of simvastatin was independently associated with a greater adjusted odds of failing to attain not only LDL-C but also non-HDL-C and ApoB goals at the very high ASCVD risk group.

Our findings provide a useful overview of dyslipidemia management and treatment outcomes in ASCVD diabetic patients stratified by gender in the Middle East. However, the study is not without limitations, it is an observational cross-sectional trial that captured only a snapshot of variables at a point in time and did not assess long-term outcomes. Missingness of important variables like statin doses, anti-diabetic and anti-hypertensive medications including diuretics as well as baseline lipid levels, which could have affected levels and consequently outcomes, is a limitation. The population studied was relatively small and considerable variability in practice patterns across the Arabian Gulf exists, and probably even among study sites, and therefore caution should be exercised when extrapolating the results to the general population.

## Conclusion

This study has demonstrated that diabetic women with ASCVD were less likely to be on optimal lipid lowering therapy compared to men and therefore to attain lipid goals proposed to prevent cardiovascular events. This clearly shows a sex gap on lipid management in the region that needs to be reduced with urgency considering the elevated prevalence of diabetes in the region and the high risk of cardiovascular and complications and mortality that diabetic women bear. Diabetic women with very high ASCVD risk status need to be aggressively treated to lower their risk of cardiovascular events.

## Abbreviations

AOR: Adjusted odds ratio
ApoB: Apolipoprotein b
ApoE: Apolipoprotein e
ASCVD: Atherosclerotic cardiovascular disease
BMI: Body mass index
CEPHEUS: Centralized pan-middle-east survey
CHD: Coronary heart disease
CI: Confidence interval
CVD: Cardiovascular disease
Hba1c: Glycated hemoglobin a1c
HDL-C: High-density lipoprotein cholesterol
LDL-C: Low-density lipoprotein cholesterol
LLDs: Lipid lowering drugs
LTAP-2 study: Lipid treatment assessment panel-2 study
NLA: National lipid association
TC: Total cholesterol
TG: Triglycerides
